# Production of cellulosic ethanol and value-added products from corn fiber

**DOI:** 10.1186/s40643-022-00573-9

**Published:** 2022-08-13

**Authors:** Yingjie Guo, Guodong Liu, Yanchun Ning, Xuezhi Li, Shiyang Hu, Jian Zhao, Yinbo Qu

**Affiliations:** 1 State Key Laboratory of Microbial Technology, Shandong University, No. 72, Binhai Road, Qingdao, 266237 Shandong China; 2 Research Institute of Jilin Petrochemical Company, PetroChina, No. 27, Zunyidong Road, Jilin City, 132021 Jilin China

**Keywords:** Corn fiber, Structure and chemical compositions, Cellulosic ethanol, Value-added products, Production

## Abstract

Corn fiber, a by-product from the corn processing industry, mainly composed of residual starch, cellulose, and hemicelluloses, is a promising raw material for producing cellulosic ethanol and value-added products due to its abundant reserves and low costs of collection and transportation. Now, several technologies for the production of cellulosic ethanol from corn fiber have been reported, such as the D3MAX process, Cellerate™ process, etc., and part of the technologies have also been used in industrial production in the United States. The ethanol yields range from 64 to 91% of the theoretical maximum, depending on different production processes. Because of the multicomponent of corn fiber and the complex structures highly substituted by a variety of side chains in hemicelluloses of corn fiber, however, there are many challenges in cellulosic ethanol production from corn fiber, such as the low conversion of hemicelluloses to fermentable sugars in enzymatic hydrolysis, high production of inhibitors during pretreatment, etc. Some technologies, including an effective pretreatment process for minimizing inhibitors production and maximizing fermentable sugars recovery, production of enzyme preparations with suitable protein compositions, and the engineering of microorganisms capable of fermenting hexose and pentose in hydrolysates and inhibitors tolerance, etc., need to be further developed. The process integration of cellulosic ethanol and value-added products also needs to be developed to improve the economic benefits of the whole process. This review summarizes the status and progresses of cellulosic ethanol production and potential value-added products from corn fiber and presents some challenges in this field at present.

## Introduction

Owing to the need to reduce greenhouse gas emissions and the ever-increasing energy demand, biofuels, such as bioethanol, as an alternative energy source, are attracting worldwide attention (Pragya et al. 2013; Rocha et al. 2014). Biofuel is a multiple objective sustainable resource that promises to substitute fossil fuels with energy from agricultural and forestry sources while providing a range of other benefits (Lovett et al. 2011). In addition, bioethanol can diversify energy supplies while contributing significantly to reducing carbon and particle emissions (Moore et al. 2017). Bioethanol is classified into three generations depending on the raw materials used in production. The first-generation bioethanol is produced from grains and starch-based feedstocks, such as sugar cane, corn, potato, and cassava. Currently, commercial bioethanol is mainly first-generation ethanol. Among them, ethanol produced from sugar crops, such as sugarcane and beet accounts for 40%, and the remaining 60% of total ethanol is produced by starch crops, such as corn (Mussatto et al. 2010; Vohra et al. 2014). Now, tens of millions cubic meters of the annual production of bioethanol still cannot meet the growing energy demand (Lennartsson et al. 2014); on the other hand, the demand for feedstocks for first-generation ethanol production requires huge cultivatable land while directly competing with the food supply (Saha and Cotta 2010; Nigam and Singh 2011; Saini et al. 2015; Searchinger et al. 2018). This problem has promoted the rise of second-generation biofuels (Puri et al. 2012). The second-generation bioethanol is produced from lignocellulosic biomass, such as crop residues, woody crops, or energy grasses, which is abundant and relatively cheap (Vohra et al. 2014; Byadgi and Kalburgi 2016; Robak and Balcerek 2020). At present, it is well known that the production of second-generation ethanol is technically feasible, but the high production costs hinder cellulosic ethanol’s fully commercial production (Sims et al. 2010), which include the raw materials cost, pretreatment cost, enzyme cost, etc. Among them, the costs of collection and transportation are important factors affecting the cost of raw materials.

As one of the easily available lignocellulosic materials, corn fiber has attracted much attention from many researchers. Corn fiber is composed of residual starch, cellulose, and hemicelluloses and makes up about 10% of the weight of corn kernel. In traditional corn ethanol facilities, corn fiber passes through the fermentation and distillation stages and ends up in the distiller’s dried grains with solubles (DDGS) (Bothast and Schlicher 2005). As a byproduct of the corn ethanol industry, it is easily collected and inexpensive for transport. According to the estimation, if the corn fiber can be economically converted into ethanol, it would increase the total corn ethanol production by up to 13% on the existing basis while improving the protein content of DDGS (Bothast and Schlicher 2005; Nair et al. 2017).

In recent years, several technologies for ethanol production from corn fiber have been reported and applied in the United States, which have increased the yield of ethanol to different degrees. For example, using a D3MAX process from D3MAX LLC (USA), the cellulose and hemicelluloses in the wet cake are hydrolyzed to fermentable sugars by dilute acid steam explosion pretreatment and enzymatic hydrolysis, which not only increases the ethanol production by 10% but also increases the protein content in DDGS to about 40% (https://www.d3maxllc.com/technology). ICM (ICM International) used Selective Milling Technology™ to further grind the granules to release more starch during ethanol production, and Fiber Separation Technology™ to separate the fiber through counterflow washing. Then both hexose and pentose from the fiber were further converted to ethanol through pretreatment, enzymatic hydrolysis, and fermentation process. Finally, the total ethanol production was increased by about 10%, and the protein content in DDGS was significantly increased (https://icminc.com/process-technologies/selective-milling-technology/). Fluid quip technologies company used Selective Grind Technology™ and Fiber By-Pass System™ to expose more starch and to shear open the germ to release more corn oil, while separating and grinding the fibers after the starch was liquefied to increase ethanol production, which resulted in a 3% increase in ethanol production and 30% increase in corn oil production (https://fluidquiptechnologies.com/proven-technologies/). Syngenta used Cellerate™ process technology to perform dilute acid pretreatment and enzymatic fermentation on the stillage. In addition, Enogen^®^ corn enzyme technology was used to deliver robust alpha-amylase enzyme directly in the grain and thus reduced liquid enzyme and chemical costs. Finally, the ethanol production increased by 6%, the protein content in DDGS increased to 40%, and the yield of corn oil was also increased (https://www.syngenta-us.com/corn/enogen/). In addition, industry players also include DuPont and Novozymes, which provide enzyme preparations for the production of corn fiber ethanol. Aiming at the different stages of corn fiber ethanol production, Novozymes introduced several enzyme preparations, including Frontia^®^, Fiberex^®^, etc. For example, the wet milling enzyme Frontia^®^ could improve the grinding capacity and increase the starch yield, while Fiberex^®^ could increase the production of ethanol and corn oil.

**Fig. 1:**
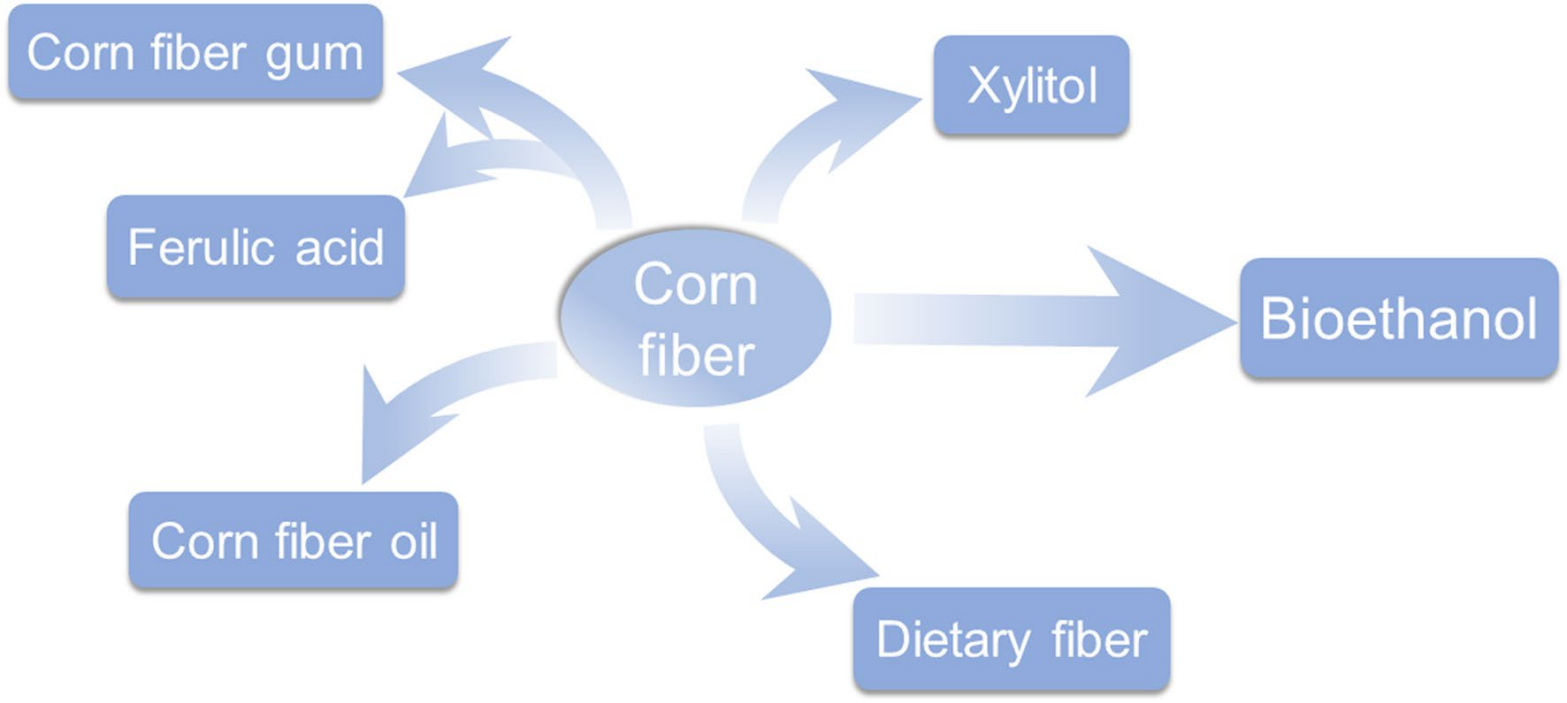
Biorefining of corn fiber to produce fuel and other value-added products

However, the current production cost of ethanol using corn fiber is still relatively high. Besides further research on the production process, the concept of biorefinery, which produces ethanol and other high-value chemicals from corn fiber, has been introduced. The other value-added products include dietary fiber, corn fiber oil, corn fiber gum, xylitol, and so on (Rose et al. 2010). This paper reviews the chemical compositions and structure of corn fiber, and the status and progress of corn fiber ethanol production technology, including the pretreatment process, enzymatic hydrolysis, enzyme system, and fermentation. Several value-added products are presented (Fig. 1), and some researches in further work are also proposed for promoting the technology development of corn fiber bioethanol.

## Chemical compositions and structure of corn fiber

Corn fiber contains approximately 70% carbohydrates, including cellulose, xylan, residual starch, and other components, such as lignin, protein, and phenolic acid (Noureddini and Byun 2010). Table 1 lists the chemical compositions of corn fiber. The total glucan content of corn fiber was similar to that of corn stover (30.6%) and corn cob (34.8%) (Eylen et al. 2011); however, unlike glucose in corn stover originates mainly from cellulose, the glucose in corn fiber is mainly present in starch (Eylen et al. 2011). The chemical compositions of corn fiber also vary according to the variety and place of production.

**Table 1:** Chemical composition (%) of corn fiber (Eylen et al. 2011)

Component	Composition (% dry weight)
Starch	16
Cellulose	14
Hemicelluloses (total)	39
Xylose	18
Arabinose	12
Galactose	3.3
Mannose + Rhamnose	2.0
Glucuronic acid	3.7
Ferulic acid (esters)	3.1
Coumaric acid (esters)	0.3
Acetic acid (esters)	3.2
Protein	10
Lignin	5.7

Cellulose, the major structural component of plant cell walls, is a linear homopolysaccharide consisting of glucose units (500–15,000) linked by β-1, 4-glucoside bonds, with cellobiose as the smallest repetitive unit (Chen 2014; Saini et al. 2015). Hydrogen bonds and van der Waals forces make this native cellulose highly crystalline, insoluble in water at high temperatures, and resistant to enzymatic hydrolysis (Chatterjee et al. 2015). Depending on its structural form, cellulose can be either ordered (crystalline) or disordered (amorphous) regions (Horn et al. 2012).

**Fig. 2:**
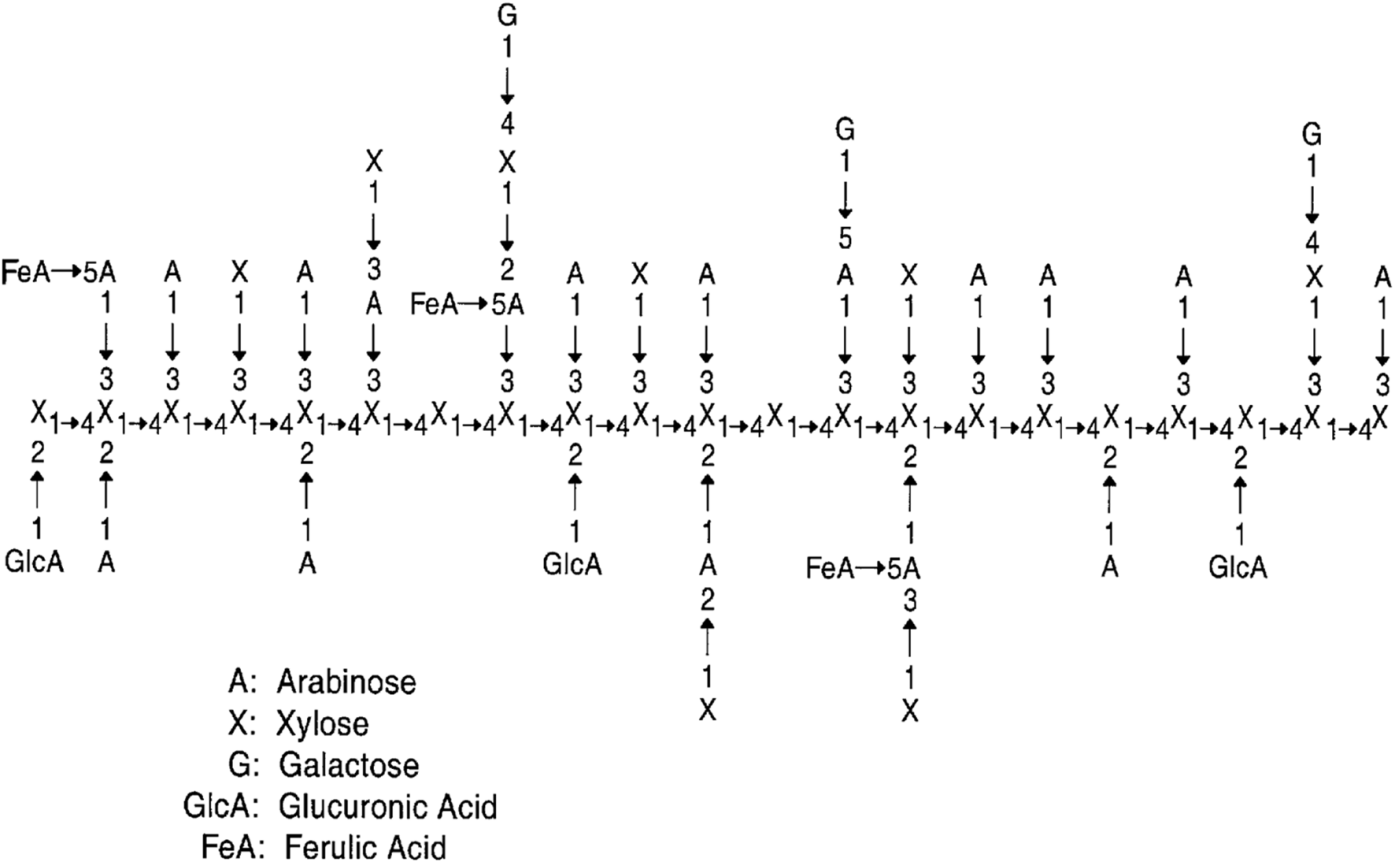
Schematic structure of the sugar moiety of heteroxylans from corn fiber (Saha 2003)

Hemicelluloses are the main component of corn fiber. Hemicelluloses are short, highly branched heteropolymers with 50–200 units consisting of different pentose sugars (e.g., d-xylose and l-arabinose), hexose (e.g., d-mannose, d-galactose, and d-glucose), and uronic acids (Saha 2003). Its acetate groups are randomly attached to the hydroxyl groups of the sugar rings with ester linkages (Saini et al. 2015). Hemicelluloses mainly include xyloglucans, xylans, mannans, and glucomannans. Compared to corn stover, corn fiber had similar xylan content (about 19% in corn stover) (Eylen et al. 2011), but significantly high contents of other hemicelluloses sugars (Table 1). Unlike wood, the hemicelluloses in corn fiber are mostly xylan with the common feature of a backbone of β-1,4-linked xylose residues (Fig. 2). This xylan usually contains many arabinose residues attached to the main chain and is called arabinoxylan and glucuronoarabinoxylan (GAX) (Scheller and Ulvskov 2010). The backbone of xylan is highly substituted by various side chains, including arabinose, 4-*O*-methyl glucuronic acid, xylose, ferulic acid, and galactose (Beri et al. 2020). Besides monosaccharide residues, there are disaccharide residues, trisaccharide residues, and even tetrasaccharide residues in the side chains. The variety of terminal sugar residues in corn fiber hemicelluloses also indicates that its side chain substitutions are very rich (Kang et al. 2019). Hemicelluloses, cellulose, and lignin are cross-linked to form a complex network structure, improving the structural stability and making hemicelluloses difficult to dissolve and be hydrolyzed (Yadav et al. 2007; Kang et al. 2019).

Lignin, found in all land plants, is a complex phenolic polymer composed of three phenylpropane units (coumarin, sinocrosinol, and coniferous alcohol) connected by carbon–carbon (C–C) and ether (C–O) linkages (Sharma et al. 2020). Lignin is mainly located between cellulose fibers, plays a role in resisting pressure, and keeps the plant’s rigid structure (de Gonzalo et al. 2016). At the same time, lignin is also a physical barrier for enzymatic hydrolysis and microbial degradation, because it is closely combined with cellulose and hemicelluloses to form a lignin–carbohydrate complex (LCC) (Bajpai 2016). Lignin also inevitably leads to the decrease of the hydrolysis yield by non-productive adsorbing cellulase, blocking the enzyme access to cellulose and reducing the cellulases activity (Lu et al. 2018; Feng et al. 2021). Compared with straw or wood (about 20%), the lignin content of corn fiber was relatively low (Bertrand et al. 2016). Phenolic acids are the most widely distributed plant non-flavonoid phenolic compounds found in free, conjugated soluble, and insoluble binding forms. They are one of the health-promoting phytochemicals found in cereals. The common phenolic acids in corn fiber include ferulic acid and *p*-coumaric acid (Menon et al. 2016). Phenolic acids have antioxidant properties and avoid cell damage caused by free radical oxidation reactions.

## Status and progress of cellulosic ethanol production technologies from corn fiber

In the traditional corn ethanol production process, the starch in corn kernels is gelatinized, liquefied, and further fermented into ethanol using simultaneous saccharification and fermentation. In this process, the recalcitrant structure of corn fiber decreases its accessibility to enzymes, resulting in most corn fiber not being converted. To effectively use the corn fiber, pretreatment is needed to destroy the structure. It is then converted into ethanol by enzymatic hydrolysis and fermentation (Kurambhatti et al. 2018; Juneja et al. 2021). Similar to the second-generation ethanol production process, bioethanol production from corn fiber mainly includes three steps, pretreatment, enzymatic hydrolysis into fermentable sugars, and ethanol fermentation.

### Pretreatment of corn fiber

The complex structure of corn fiber makes it difficult to be directly hydrolyzed by enzymes, so it needs to be pretreated to improve the enzymatic hydrolysis efficiency. Pretreatment could promote the hydrolysis of cellulosic substances by destroying cellulose’s crystallinity, and removing lignin and/or hemicelluloses (Mankar et al. 2021). Effective pretreatment must improve enzymatic hydrolysis, reduce carbohydrate loss, and prevent the formation of byproducts that may inhibit subsequent enzymatic hydrolysis and fermentation steps, such as furfural, hydroxymethylfurfural (HMF), phenolic acids, etc. (Qureshi et al. 2008; Kurambhatti et al. 2018). Physical, physical–chemical, chemical and biological processes have been extensively studied for the pretreatment of lignocellulosic materials (Hendriks and Zeeman 2009; Alvira et al. 2010). This section briefly describes the main types of pretreatments related to corn fiber.

#### Physical pretreatment

The purpose of physical pretreatments, for example, milling, grinding, and extrusion, is to increase the surface area of the cellulosic substrate and reduce its particle size and cellulose crystallinity (Harun et al. 2011; Mankar et al. 2021). During physical pretreatment, mechanical tools and techniques, such as milling machines, grinders, ball mills, etc., are usually used to cut or grind the corn fiber, thereby releasing the starch bound to it, realizing the homogenization and dispersion of the material, and making the enzymatic hydrolysis rate significantly increase. However, physical pretreatment such as grinding is an energy-intensive process and is not economically feasible commercially.

Lin and Ryu (2014) applied thermo-mechanical extrusion to pretreat corn fiber to reduce particle size (PS) and evaluated their effects on the bioconversion of corn fiber into fermentable sugars and ethanol production. The extrusion was conducted at 140 °C, raw and the extruded corn fiber were separated into three different PSs (1 > PS ≥ 0.5, 0.5 > PS ≥ 0.3, and 0.3 > PS ≥ 0.15 mm) with a wire sieve. The results showed that thermo-mechanical extrusion pretreatment and PS reduction significantly increased the yields of total sugars and reducing sugars, thereby improving the fermentation performance, increasing the ethanol yield and the conversion rate of corn fiber. Heating during extrusion accelerated the degradation of corn fiber structure and the release of hemicelluloses soluble compounds, and reduced total content of phenolics degraded from pretreated corn fiber. The decrease in total phenol content may be due to the decomposition of phenolic compounds at high extrusion temperature or changes in molecular structure (Sharma et al. 2012). However, unlike the corn fiber with large PS, using the corn fiber at 0.3 > PS ≥ 0.15 mm did not reduce total phenol content after thermo-mechanical extrusion pretreatment, which may be due to the excessive reduction in size that caused a large number of phenolic components to be released. This was also similar to the results reported by Kim et al. (2017), indicating that a large degree of size reduction is not desirable. Singkhornart et al. (2013) combined extrusion pretreatment with a chemical pretreatment, through, respectively, performed sulfuric acid pretreatment and sodium hydroxide pretreatment on the extruded corn fiber, further increasing the reducing sugar content in the pretreatment filtrate. This indicated the extrusion technology was an efficient tool for the dispersion and reduction of particle size of corn fiber.

#### Physical–chemical pretreatment

The physicochemical pretreatments include steam explosion pretreatment, ammonia fiber explosion (AFEX) pretreatment, and liquid hot water (LHW) pretreatment.

Steam explosion pretreatment is often used to treat lignocellulosic biomass, and its energy consumption and cost are relatively moderate (Conde-Mejía et al. 2012). In the steam explosion, high temperature (160–260 °C) and high-pressure steam are used to heat the biomass for a few seconds to a few minutes. At the end of the reaction, the pressure drops suddenly, making the material explode and decompress. During this process, hemicelluloses are partially degraded, and the structure of cellulose becomes loose due to the destruction of the crystalline area, which is conducive to enzymatic hydrolysis (Taherzadeh and Karimi 2008). Bura et al. (2002) used a batch reactor to steam explode corn fiber at various degrees of severity. The corn fiber was impregnated overnight with anhydrous SO_2_ in plastic bags and then put into the reactor for the steam explosion. The results indicated that maximum sugar yield (81%) was recovered from corn fiber pretreated at 190 °C for 5 min with 6% SO_2_. Subsequently, Bura et al. (2003) studied the effect of each parameter in the steam explosion pretreatment process on the sugar yield through the response surface model. It was found that all the parameters studied in the steam explosion pretreatment process had significant effects on the sugar recovery, inhibitory formation, enzymatic conversion efficiency, and fermentation capacity of yeast. Steam explosion pretreatment uses limited chemicals and requires low energy input, but its high temperature and pressure can also lead to the formation of fermentation inhibitors and further degradation of products.

AFEX pretreatment is an alkaline physicochemical pretreatment process, in which, lignocellulose is pretreated with liquid ammonia–water mixtures (Balat et al. 2008; Behera et al. 2014). In this process, biomass is exposed to liquid ammonia at a moderate temperature. After a brief residence time under set pressure, the pressure decreases suddenly, which leads to the cracking of the lignin–carbohydrate complex and the physical destruction of biomass fiber. AFEX pretreatment has the advantages of lower moisture content and lower formation of sugar degradation products. Hanchar et al. (2007) mixed corn fiber with an equal weight of liquid ammonia at 90 °C and released the pressure after holding it for 30 min. After AFEX treatment, 84% of glucan conversion rate was obtained after 24 h of enzymatic hydrolysis of corn fiber, and xylan and arabinan were also dissolved in the hydrolysate. Corn fiber gum was precipitated by adding ethanol, and then corn fiber gum was hydrolyzed into monosaccharides with dilute acid. This indicated that although AFEX pretreatment can effectively hydrolyze the cellulose in corn fiber, it is difficult to destroy the network structure of hemicelluloses in corn fiber. Another problem of AFEX pretreatment is the cost of ammonia and the need for recovery technology.

In the LHW, lignocellulosic materials are treated using water at high pressure and high temperature (160–220 °C) for a certain time. By controlling pH between 4 and 7, inhibitor formation and sugar degradation could be effectively prevented during LHW pretreatment (Hendriks and Zeeman 2009). LHW pretreatment can dissolve more than 80% of hemicelluloses in biomass and expand cellulose’s accessible and susceptible surface area by removing hemicelluloses, making it easier to be used by hydrolases (Li et al. 2014). Allen et al. (2001) fractionated corn fiber by treatment with hot liquid water at low solids loadings (5–10%) at 210–220 °C for 2 min, the solubilization of corn fiber reached 54% at 215 °C, and most of the pentosans in the hydrolysate were in the form of oligomers. The glucan-rich lignocellulose residue from this hot liquid water fractionation was reactive to enzymatic hydrolysis and fermentation, exhibiting higher conversion of glucan to ethanol (86%) than that with untreated corn fiber (64%), but the liquid fraction from the process did not inhibit the final yield of glucose fermentation, while the fermentation rate was reduced. Juneja et al. (2021) performed a two-step pretreatment on corn fiber separated from whole stillage, including LHW pretreatment and disk milling. The best conditions were that corn fiber was treated by liquid hot water pretreatment under 25% of solids content at 180 °C for 10 min, followed by three cycles of disk milling. Under these conditions, glucose, xylose, and arabinose yields were 94.9%, 74.2%, and 66.3%, respectively. After fermentation, the protein content in the residual solids (DDGS) reached 52.05%. LHW is a mild pretreatment without chemicals addition, which can minimize the formation of inhibitory products, but the conversion of cellulose in the solid substrate from LHW pretreatment is lower than that from dilute acid pretreatment during enzymatic hydrolysis. To obtain similar enzymatic digestibility of substrate, longer residence time and higher temperature are required. The LHW pretreatment is regarded as energy-demanding because of higher pressures and large amounts of water supplied to the system.

#### Chemical pretreatment

Chemical pretreatment uses a chemical reaction to change the recalcitrant structure of lignocellulosic materials. The commonly used chemicals for the pretreatment of corn fiber include acid, alkali, and peroxide. Chemical pretreatments have been observed to be more effective than non-chemical pretreatments; however, they also have limitations, such as inhibiting compounds formation, neutralization requirements of downstream processes, and environmental issues.

Acid pretreatment is the most commonly used chemical method for lignocellulosic materials, and sulfuric acid is one of the best solvents for hemicelluloses dissolution. During the acid pretreatment, hemicelluloses are hydrolyzed into soluble sugars, making cellulose more accessible. Eylen et al. (2011) reported that, as the pretreatment conditions became severe, the solid residues became less after pretreatment of corn fiber with dilute acid, and the concentrations of monosaccharides in the pretreatment liquid increased, but there were still a large number of oligosaccharides in hydrolysates. Granados-Arvizu et al. (2017) optimized dilute sulfuric acid pretreatment of corn peel by response surface methodology to maximize the yield of reducing sugars. This indicated that under the optimal conditions (sulfuric acid concentration 3.43% (w/v), solid concentration 20% (w/v), 121 °C for 22.3 min), almost all hemicelluloses in corn pericarp were solubilized, and 78.9 g/L ± 1.9 g/L of total reducing sugars, and 11.7 g/L ± 0.8 g/L of glucose were obtained. However, the disadvantages of the dilute acid pretreatment are that it can corrode equipment and produce inhibitors, such as furfural and HMF (Jönsson and Martín 2016). Therefore, dilute acid pretreatment is usually performed at low temperatures to reduce the cost and the formation of inhibitors (Bhutto et al. 2017). For example, Noureddini and Byun (2010) reported that using dilute sulfuric acid to pretreat DDGS and corn fiber, the highest yield of monomeric sugars was observed at 140 °C, but it was also accompanied by more furfural formation. When reducing the pretreatment temperature to 120 °C, the furfural concentration in hydrolysates was significantly decreased. Inhibitors in the hydrolysate are usually removed by water washing or overliming, but this can cause problems, such as wastewater and the loss of sugars. Different from this, Zhang et al. (2021) adopted biodetoxification by inoculating *Paecilomyces variotii* to remove inhibitors produced by citric acid pretreatment, and the ethanol concentration reached 70.2 g/L using semi-SSF at 25% of solid content, and ethanol production reached 0.280 g/g initial corn fiber. Guo et al. (2022) optimized the pretreatment conditions by response surface methodology to obtain high yield fermentable sugars and reduce the formation of inhibitors, the mixture of solid and hydrolysates obtained by dilute acid pretreatment of corn fiber was directly used for semi-simultaneous saccharification and fermentation without detoxification, and the ethanol yield reached about 81% of the theoretical yield.

Alkali pretreatment destroys the cell walls structure of lignocellulosic material by dissolving part hemicelluloses and lignin, hydrolyzing uronic and acetic acid esters, and reducing cellulose crystallinity, thereby promoting enzymatic degradation (Taherzadeh and Karimi 2008; Hendriks and Zeeman 2009). Shrestha et al. (2010) pretreated corn fiber with 2% (w/w) NaOH at 30 °C for 2 h to study the effect of mild alkali pretreatment on saccharification by white-rot fungus and sequential simultaneous saccharification and fermentation (SSF) to ethanol and found that compared with unpretreated corn fiber, mild alkaline pretreatment resulted in higher glucose yield and similar ethanol production. Myat and Ryu (2014) used thermo-mechanical extrusion followed by NaOH of 0.75% (w/v) to treat destarched corn fiber, and found that the combined method pretreatment significantly decreased lignin content, crystallinity index and cellulose polymerization degree, thereby effectively improved enzymatic saccharification and significantly increased ethanol yield (from 8.64 to 35.13 g/L). However, a significant disadvantage of alkali pretreatment is that the alkali will be converted into unrecoverable salts and/or the salts will be mixed into the biomass during the pretreatment reaction. Therefore, treating large amounts of salts has become a challenging problem in alkaline pretreatment.

#### Biological pretreatment

Biological pretreatment is an environmentally friendly pretreatment method that applies microorganisms, especially fungi, such as white rot fungi, brown rot fungi, and soft rot fungi, to convert lignocellulosic materials into more accessible components for hydrolysis (Singh et al. 2008). Compared with other pretreatments, biological pretreatment is low energy consumption and mild conditions but a very slow process (Wyman et al. 2005). Shrestha et al. (2008) mixed corn fiber with *Phanerochaete chrysosporium* fungal pellets for pretreating corn fiber by solid fermentation, and the weight loss rate of biomass and reduction rate of lignin after 3 days of cultivation were 34% and 41%, respectively. After aerobic solid-substrate fermentation of 3 days and anaerobic static submerged culture of 6 days, the ethanol yield was 1.7 g ethanol/100 g corn fiber. The low ethanol yield indicated incomplete bioconversion. Subsequently, they combined the solid-state fermentation of corn fiber by white rot fungi or brown rot fungi with simultaneous saccharification and fermentation, and the highest ethanol yield reached 8.6 g ethanol/100 g initial corn fiber, which was equivalent to 35% of the theoretical ethanol yield of starch and cellulose in corn fiber. However, its entire process time (15 days) was very long (Shrestha et al. 2009), making it difficult to realize industrial production.

As mentioned above, compared to other lignocellulosic biomass, such as corn stover and wood, the prominent features of corn fiber are that corn fiber contains a higher proportion of hemicelluloses, more types of hemicelluloses sugars, more complex structure of hemicelluloses, and very low content of lignin. Thus, it is required to pay more attention to the degradation of hemicelluloses structure during pretreatment for decreasing hemicelluloses sugars being further degraded into inhibitors as well as improving the enzymatic digestibility of corn fiber.

### Enzyme used for hydrolysis of corn fiber

After the pretreatment step, enzymes need to be added to hydrolyze the lignocellulosic biomass to release fermentable sugars. The complex components and structure of corn fiber require a complex enzyme system to achieve effective hydrolysis, and the enzyme mixture was composed of several enzyme entities, such as cellulases, hemicellulases, and accessory proteins.

#### Cellulase

Cellulase refers to the general name of the multi-component enzymes involved in the degradation of cellulose, which mainly includes three major categories: endoglucanase (EC 3.2.1.4), exoglucanase or cellobiohydrolase (EC 3.2.1.91) and β-glucosidase (EC 3.2.1.21) (Zhang et al. 2006). The above three enzymes can synergistically degrade cellulose: endoglucanase can randomly cleave the cellulose chain in the amorphous region of cellulose to reduce the polymerization degree of cellulose and form new reaction sites for exoglucanase; exoglucanase attacks the end of the cellulose chain to produce cellobiose; β-glucosidase hydrolyzes cellobiose into glucose. In the degradation of cellulose, the three hydrolysis processes co-occur. The primary enzymatic hydrolysis occurs on the surface of the solid substrate, here, endoglucanase and exoglucanase hydrolyze cellulose and release low-polymerization cello-oligosaccharides into the liquid phase, which is the rate-limiting step of the entire cellulose hydrolysis, followed by secondary hydrolysis by β-glucosidase in the liquid phase, mainly involving the hydrolysis of cellobiose to glucose. However, most cellulases produced by fungi lack the activity of β-glucosidase, and the accumulation of cellobiose results in the inhibition on cellulase activity, but the feedback inhibition of cellobiose could be relieved by overexpressing homologous or heterologous β-glucosidase in cellulase producing strain, thus enhancing the hydrolysis efficiency of cellulase mixtures (Yao et al. 2016; Gao et al. 2017).

#### Hemicellulases

Hemicelluloses have very complex compositions, most of which are based on xylan as the main chain, and the side chains have various substituents, so the enzymatic hydrolysis of hemicelluloses requires a variety of enzymes (Sun et al. 2012). The core enzymes used for the degradation of the xylan skeleton mainly include endo-β-1,4-xylanase (EC 3.2.1.8) and β-xylosidase (EC 3.2.1.37) (Méndez-Líter et al. 2021). Endo-β-1,4-xylanase can break the β-1,4 glycoside bonds on the xylan skeleton, thereby reducing the polymerization degree of xylan. β-xylosidase can release β-xylose by acting on the non-reductive end of hypoxic oligosaccharides. In addition, some accessory enzymes are needed to cleave the side chains, including α-L-arabinofuranosidase (EC 3.2.1.55), α-glucuronidase (EC 3.2.1.139), acetylxylan esterase (EC 3.1.1.72), ferulic acid esterase (EC 3.1.1.73), α-galactosidase (EC 3.2.1.22) and α-xylosidases (EC 3.2.1.177) (Saha, 2003). α-l-Arabinofuranosidases hydrolyze the linkages between the α-l-arabinofuranosyl substituent and the xylopyranose unit of the backbone, and they act from the non-reducing end of the target linkages to release the arabinofuranose (Poria et al. 2020). α-Glucuronidases hydrolyze the glycosidic bonds between glucuronic acid or 4-*O*-methylglucuronic acid and the xylopyranose unit of the xylan backbone (Juturu and Wu 2014). Acetylxylan esterases catalyze the hydrolysis of ester linkages between xylopyranose residues and their acetyl substituents and belong to the carbohydrate esterases (CE) superfamily (Kameshwar and Qin 2018). Feruloyl esterases hydrolyze ferulic acid residues esterified to certain arabinoses in the side chains of xylan and are grouped exclusively in family CE1 (Oliveira et al. 2019). α-Galactosidase of GH95 hydrolyzes α-L-Gal-1,2-Xyl linkages on side branches of corn glucuronoarabinoxylan to release l-galactose. α-Xylosidases act on xyloglucan and hydrolyze α-d-xylopyranosyl-1,6-d-glucopyranose into d-xylose and d-glucose (Matsuzawa et al. 2020).

Due to the complex structure and sugar compositions of hemicelluloses in corn fiber, the enzyme system with multiple enzymes is required to synergistically degrade the hemicelluloses in corn fiber. For example, a variety of α-arabinofuranosidases (GH43, GH51, GH54, GH62), α-galactosidase, β-galactosidase, α-glucuronidase, mannosidase, etc. sequentially hydrolyze and remove the sugar residues in the side chain, and then xylanase and xylosidase break the xylan skeleton to form monosaccharides.

#### Accessory proteins

In addition to cellulases and hemicellulases, some accessory proteins can also promote the hydrolysis of corn fiber by breaking down the structure of cellulose, increasing the accessibility and looseness of cellulose, and further enhancing the hydrolysis performance of various enzymes against the substrate (Yang et al. 2018). For example, LPMOs (lytic polysaccharide monooxygenases) are copper-dependent enzymes that cleave glycosidic bonds in cellulose fibers in an oxidative manner, but these glycosidic bonds can’t be degraded by cellulase. It has been considered a booster for biomass deconstruction (Singhania et al. 2021). When the LPMOs act in synergism with cellulase components, the number of soluble fragments increase dramatically, thus significantly improving the hydrolysis efficiency of cellulase. Swollenin is a kind of expansin-like protein secreted by various microorganisms, and it can loosen cellulose fibers and increase porosity by breaking the hydrogen bonds in the cellulose microfiber network, thereby enhancing the accessibility of cellulose. Swollenin is active on substrates containing β-1,4 glycosidic bonds, such as carboxymethyl cellulose, hydroxyethyl cellulose, and β-glucan (Meng et al. 2020).

#### Cellulase producing strain

Cellulase can be produced by a wide range of organisms, including bacteria, actinomycetes, filamentous fungi, and plants and animals (Kuhad et al. 2011). Among these organisms, filamentous fungi are the most prominent, including *Trichoderma*, *Penicillium*, and *Aspergillus* (Druzhinina and Kubicek 2017; Qian et al. 2017; Liu and Qu 2019).

*Trichoderma reesei* is the most widely used strain for cellulase production and has been used by many leading enzyme production groups in the world, such as Novozymes (Bischof et al. 2016). The types and numbers of lignocellulose-degrading enzyme genes of *T. reesei* are relatively few, and many of which are considered to be derived from horizontal gene transfer, but the cellulase from *T. reesei* has a strong ability to degrade crystalline cellulose (Druzhinina and Kubicek 2016; Druzhinina et al. 2018).

There are many types of *Penicillium* that can secrete cellulase, among which *Penicillium oxalicum* is representative (Liu et al. 2013a). Compared with *T. reesei*, *P. oxalicum* can secrete the cellulase enzyme containing rich hemicellulase species, and the more balanced enzyme compositions may be more favorable for hydrolyzing corn fiber (Liu et al. 2013b; Gong et al. 2015; Gao et al. 2021). In addition, *Penicillium* has a faster growth rate than *Trichoderma*, and its enzyme has good stability during enzymatic hydrolysis. All these make *Penicillium* have certain advantages and become a good choice for developing cellulase preparations for producing cellulosic ethanol from corn fiber.

*Aspergillus niger*, one of the most important cell factories in biotechnology research, has the advantages of high protein yield, high protein secretion efficiency, and high safety of synthetic products (Cairns et al. 2018). The enzyme mixture from *Aspergillus* usually has high activities of amylase, hemicellulase, pectinase, and β-glucosidase, which are conducive to degrading the components, such as starch, hemicelluloses, pectin, cello-oligosaccharides in biomass, but *A. niger* is generally poor utilization of crystalline cellulose (Coutinho et al. 2009).

*Neurospora crassa* is a model organism that can produce cellulase using microcrystalline cellulose as a carbon source, and the number of cellulase genes far exceed that of *T. reesei* (Roche et al. 2014). However, there is still a big gap between the level of enzyme production and the requirements of industrial production.

The high cost of enzyme is a key bottleneck for the commercial production of cellulosic biofuels (Liu et al. 2016), and optimizing the compositions of cellulase is the main strategy to reduce the enzyme cost by enhancing the hydrolysis efficiency of cellulase (Harris et al. 2014; Li et al. 2017). Although fungi have a better ability to produce cellulase, up to now, almost no single fungus can produce the cellulase system with all the enzyme components required for lignocellulosic biomass degradation. Common methods for optimizing the enzyme system include overexpression of native or heterologous enzyme genes, the addition of exogenous components, such as auxiliary proteins, enhancement of the transcription of lignocellulolytic enzyme-coding genes, and the compounding of multiple enzyme systems (Yao et al. 2016; Du et al. 2018; Qu et al. 2018; Gao et al. 2021). For example, by combinatorial engineering of three transcriptional activators ClrB, XlnR, and AraR in *P. oxalicum* strain M12, Gao et al. (2021) generated a strain MCAX with 3.1- to 51.0-fold increases in lignocellulolytic enzyme production compared with the parent strain M12. In addition, the fermentation liquid produced from MCAX strain showed 3.1-, 47.5-, 51.0-, 40.6-, and 4.4-fold increases in the volumetric activities of cellulase (FPase), α-l-arabinofuranosidase, α-galactosidase, xylanase, and β-xylosidase, respectively, relative to that from the strain M12. Using the cellulase from the MCAX strain, the release of fermentable sugars from corn fiber was significantly increased compared to the cellulase of the M12 at the same protein dose. Zhang et al. (2022) constructed the engineering strain Δ4*cel*OE*xyr*1 by overexpressing XYR1, a master transactivator controlling (hemi)cellulase gene expression, in *T. reesei* QM9414-∆*pyr*4 lacking four main cellulase-encoding genes, and found that the activities of xylanase, arabinofuranosidase, and mannanase in the enzyme solution produced from the engineering strain were increased. Using the cellulase from the strain Δ4*cel*OE*xyr*1*,* the final yields of xylose and arabinose were increased by 135% and 65%, respectively, compared to the parent strain *T. reesei* Q9414-Δ*pyr*4. However, the conversion efficiency of xylose and arabinose in corn fiber were both less than 55% for these two strains.

At present, there is no mature enzyme system or commercial enzyme preparation that can completely hydrolyze corn fiber. It was reported that Novozymes has developed an enzyme preparation called Fiberex^®^ 2.5, which was uniquely able to break down important layers of the fiber, thus enhancing cellulose conversion while releasing more corn oil and residual starch. Although ethanol production was increased, only roughly 60% of the fiber was converted.

### Fermentation of ethanol

#### Microorganism for ethanol fermentation of corn fiber

The corn fiber hydrolysates are mainly composed of glucose, xylose, arabinose, galactose, etc. Among them, glucose, xylose, and arabinose are the main sugars. In nature, there are a variety of microorganisms that can produce ethanol through sugars fermentation, including yeast, such as *Saccharomyces cerevisiae*, bacteria, such as *Clostridium thermohydrosulfuricum*, *Zymomonas mobilis*, and fungi, such as *Fusarium oxysporum* and *N. crassa* (Dien et al. 2003). However, these traditional ethanol fermentation strains can only use hexose but not pentose because of lacking the upstream pathway of pentose metabolism. Therefore, it is necessary to modify these strains through genetic engineering for converting the pentoses to ethanol.

##### S. cerevisiae

**Fig. 3:**
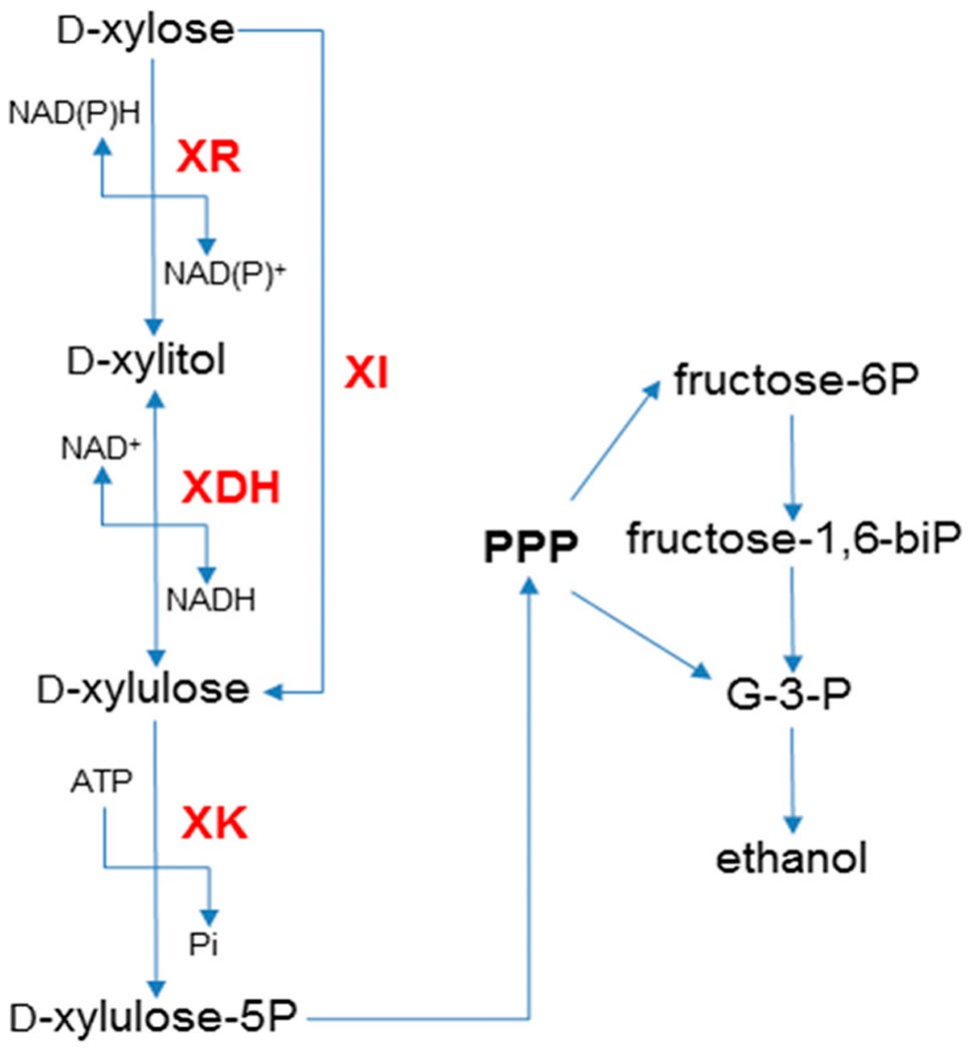
Xylose fermentation in *S. cerevisiae*. The fungal pathway uses xylose reductase (XR) and xylitol dehydrogenase (XDH), whereas the bacterial pathway uses xylose isomerase (XI). Both pathways produce d-xylulose which is converted to d-xylulose-5P by endogenous xylulokinase (XK). d-Xylulose-5P then enters the pentose phosphate pathway (PPP), where it is further metabolized to form ethanol under appropriate conditions. Arrows indicate the direction of the chemical reactions (Moysés et al. 2016)

*S. cerevisiae* has high ethanol tolerance, low pH tolerance, insensitivity to bacteriophage infection, and the ability to ferment under strictly anaerobic conditions, which makes it more advantageous than other bacteria. At present, *S. cerevisiae* is still the preferred microorganism for fuel ethanol production. As it cannot effectively convert xylose to ethanol, two metabolic pathways have been introduced into *S. cerevisiae* (Fig. 3), including the xylose oxidoreductase pathway (XR/XDH) derived from *Pichia stipitis* and xylose isomerase pathway (XI) derived from anaerobic microorganisms (Gao et al. 2019). Through two different pathways, xylose is converted to 5-xylulose, and then xylulose is phosphorylated to 5P-xylulose, which is further metabolized through the pentose phosphate pathway and glycolysis. In the engineered *S. cerevisiae* strains expressing the xylose oxidoreductase pathway, the xylose consumption rate and ethanol productivity were relatively high, but by-products such as xylitol, glycerol, and acetate were also accumulated (Kwak and Jin 2017). In the engineered *S. cerevisiae* strains expressing the xylose isomerase pathway, although the accumulation of by-products was small, the rates of yeast growth and xylose fermentation were slow (Cai et al. 2012; Li et al. 2016). However, the simple introduction of the XR/XDH or XI pathways is insufficient to ensure that recombinant yeast could ferment xylose effectively. According to the different pathways, optimizing the engineered *S. cerevisiae* strains through genetic modification, evolutionary engineering, recombinant metabolic pathways, etc. are expected to effectively reduce the production of by-products and increase the ethanol yield (Zhang et al. 2010; Moysés et al. 2016; Jeong et al. 2020). However, up to now, to improve the ability of *S. cerevisiae* to metabolize xylose, there are still many metabolic bottlenecks that need to be resolved, such as the xylose uptake, xylitol formation, improvement of catalytic efficiency of xylose isomerases, and increased specific growth in xylose.

##### Z. mobilis

**Fig. 4:**
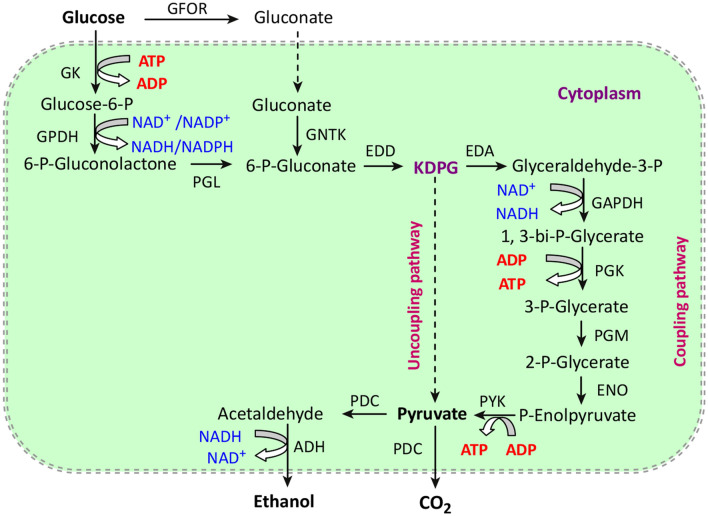
Entner–Doudoroff (ED) pathway for ethanol fermentation in *Z. mobilis*. *ADH* alcohol dehydrogenase; *EDA* 2-keto-3-deoxy-gluconate aldolase; *EDD* 6-phosphogluconate (Xia et al. 2019)

*Z. mobilis* is an industrially important ethanol-producing strain with a high specific sugar uptake rate, low pH preference, high tolerance to sugar and ethanol, and high fermentation rate (Ofosu-Appiah et al. 2016). *Z. mobilis* metabolizes sugar through the Entner–Doudoroff (ED) with less ATP while making more sugar to be used for ethanol production (Fig. 4). However, *Z. mobilis* cannot ferment C5 sugars, such as xylose natively. Through heterologous expression of genes encoding xylose isomerase (*xylA*), xylose kinase (*xyylb*), transaldolase (*tal*), and transketolase (*tktA*), *Z. mobilis* that can utilize xylose has been developed (Zhang et al. 1995). Dunn and Rao (2014) heterologously expressed the xylose-specific transporter XlyE of *Escherichia coli* in *Z. mobilis*, which facilitated xylose transport and improved the utilization of xylose in the high concentration glucose–xylose mixed medium. However, now, xylose metabolism is still far slower than glucose metabolism for the engineered *Z. mobilis*. A similar strategy was also applied to engineer *Z. mobilis* to make it has the ability of arabinose metabolism (Deanda et al. 1996). However, the application of *Z. mobilis* also has many problems, such as the narrow substrate spectrum, the competition of alternative pathways, and the cytotoxic effects of ethanol and inhibitors. Wang et al. (2017) co-expressed the alcohol dehydrogenase gene and the transhydrogenase gene in *Z. mobilis*, which enhanced the conversion rate of furfural and HMF and accelerated ethanol fermentability from the lignocellulosic hydrolysate. Wang et al. (2013a, b) shut down the metabolic pathway of sorbitol by inactivating the *gfo* gene encoding glucose–fructose oxidoreductase (GFOR), thereby increasing the growth rate of *Z. mobilis* and ethanol production. In addition, domestication, continuous cultivation, multiple strains working synergistically, and other microbial metabolism engineering strategies also positively affect the development of more robust *Z. mobilis* strains for the efficient production of ethanol from lignocellulosic biomass.

##### E. coli

*E. coli* is a model strain used for genetic modification. By heterologous expressing pyruvate decarboxylase and alcohol dehydrogenase II from *Z. mobilis* in *E. coli*, an engineered *E. coli* strain capable of producing ethanol was obtained (Ohta et al. 1991). Sanny et al. (2010) transformed the *E. coli* strain FBR5 with the hemoglobin gene of *Vitreoscilla* and obtained a construct that produced more ethanol than the parental strain FBR5 when grown in glucose and xylose. Some strategies have been used to improve the tolerance of *E. coli* to ethanol and inhibitors produced from the pretreatment process. For example, Wang et al. (2008) isolated ethanol-tolerant mutant from parent strain by enrichment method and then heterologously expressed ethanol alcohol dehydrogenase II (*adhB*) and pyruvate decarboxylase (*pdc*) genes of *Z. mobilis* to obtain ethanol-tolerant recombinant strain. In addition, by heterologous expressing beneficial alcohol tolerance genes such as GroESL from *Clostridium acetobutylicum* and phasins (polyhydroxyalkanoate granule-associated protein) from *Azotobacter* sp. in *E. coli*, the tolerance of *E. coli* to alcohol was also improved (Zingaro and Papoutsakis 2013; Mezzina et al. 2017). The furfural reductase in *E. coli* can convert furfural and HMF into less toxic alcohols, and the concentration of NADPH plays a vital role in the activity of furfural reductase (Chong et al. 2013). By knocking out the oxidoreductase genes *yqhD* and *dkgA* of NADPH in *E. coli*, Ibraheem and Ndimba (2013) increased the availability of NADPH to furfural reductase, thereby increasing the tolerance of *E. coli* to furfural and HMF. Although the genetic modification of *E. coli* has been carried out for many years, there are still some problems for these engineered strains, for example, the slow utilization rate of pentose, the slow growth rate, etc.

#### Ethanol production

The current common ethanol production processes include separate hydrolysis and fermentation (SHF), simultaneous saccharification and fermentation (SSF), semi-simultaneous saccharification and fermentation (S-SSF), and consolidated bioprocessing (CBP).

##### SHF process

SHF is a process of enzymatic hydrolysis and fermentation in sequence (Galbe et al. 2011). In this process, enzymatic hydrolysis is first performed under the optimum temperature of the saccharifying enzyme, subsequently, yeast is added to ferment the saccharification solution. Because enzymatic hydrolysis and fermentation can be conducted separately under their respective optimal conditions, therefore, results in high enzyme efficiency and ethanol productivity. In addition, in the SHF process, as the fermentation used liquid broth separated from enzymatic hydrolysis, thus the yeast after fermentation can also be recovered by filtration or centrifugation. The main disadvantage of SHF is that cellulase is inhibited by the hydrolysis products, such as oligosaccharides and glucose, that is, the productivity of the process decreases with increasing sugar concentration. In addition, after enzymatic hydrolysis, there will be a loss of sugar during the separation of solids and liquids.

##### SSF process

SSF is a process that combines enzymatic hydrolysis and fermentation, that is, both the enzymes and yeast are added simultaneously into the same fermentor with the cellulosic substrate for allowing a single-step production of ethanol (Marulanda et al. 2019). Compared with SHF, SSF uses a single vessel for saccharification and fermentation, which reduces residence time and process devices cost. At the same time, the presence of ethanol in the fermentation broth reduces the chance of contamination in the system, and the end-product inhibition of cellulase is significantly reduced because of fermentation in time. Liu et al. (2014) performed SSF and SHF of steam-exploded corn stover, and the results showed that compared with SHF, the conversion rate of glucan and the yield of ethanol in SSF increased by 13.6% and 18.7%, respectively. The disadvantage of SSF is that the temperature of the entire process is lower than the optimum action temperature of the enzyme, which may decrease enzymatic hydrolysis efficiency, thus resulting in the requirement for more enzymes. Second, it is not easy to recover yeast because of many insoluble solids in the fermentation system when using lignocellulosic biomass as feedstock.

##### S-SSF process

**Fig. 5:**
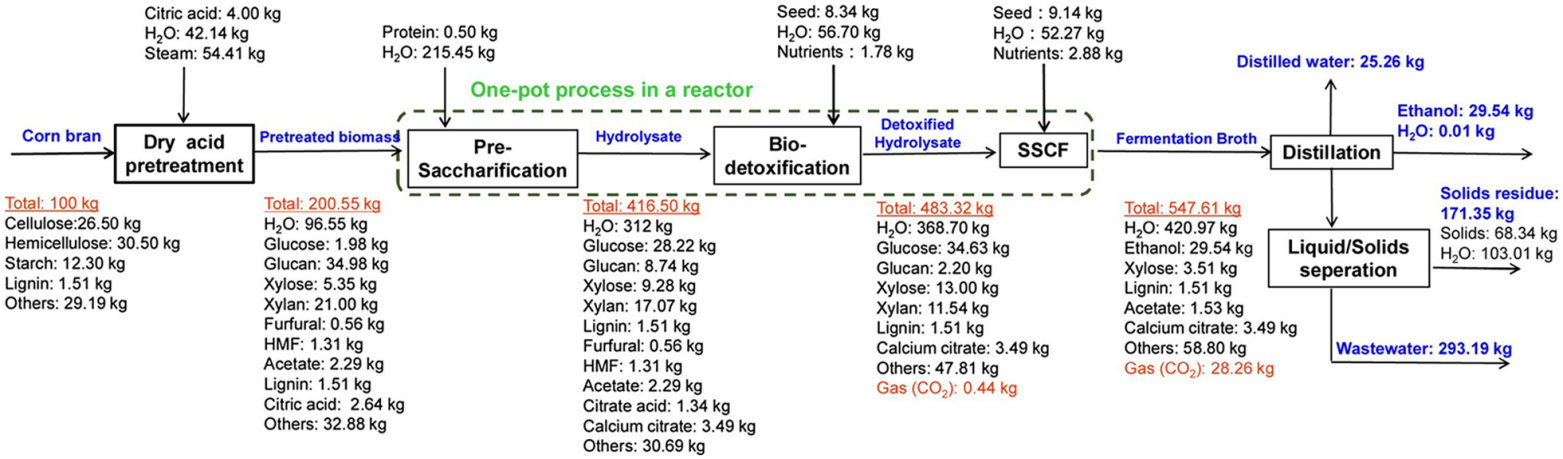
Feedstock mass balance of cellulosic ethanol production from corn fiber. The steps of pre-saccharification, biodetoxification and SSCF were carried out in the same reactor, which were considered as continuous process (Zhang et al. 2021)

In the S-SSF process, a short pre-hydrolysis of biomass is carried out before SSF under the optimal conditions of enzymatic hydrolysis, followed by SSF at a lower temperature to stimulate the vitality of microorganisms, which will promote ethanol fermentation. It is expected to combine the advantages of SSF and SHF and can obtain high productivity and ethanol yield by optimizing the suitable pre-hydrolysis process. Shen and Agblevor (2010) used a highly refined standard cellulose as a substrate and studied four different cases under the same total process time (enzymatic hydrolysis and fermentation), that is, 24 h pre-hydrolysis + 48 h SSF (S-SSF 24), 12 h pre-hydrolysis + 60 h SSF (S-SSF 12), 72 h SSF, and 48 h hydrolysis + 24 h fermentation (SHF). The results indicated that the S-SSF gave a higher yield and productivity of ethanol than the SSF and the SHF when a suitable pre-hydrolysis period is selected, in which the highest ethanol production and output were obtained using the S-SSF 24 process. Zhang et al. (2021) used corn bran as raw material, and after pretreatment, detoxification, and S-SSF, the final ethanol yield was 29.54 kg ethanol/100 kg corn bran. The feedstock mass balance for the production of cellulosic ethanol was shown in Fig. 5.

In the ethanol fermentation process, the high substrate concentration increases the amounts of inhibitory substances, thereby affecting the process efficiency of enzymatic hydrolysis and fermentation. By adopting the fed-batch S-SSF process, the substrate was added several times during the pre-hydrolysis stage of S-SSF to increase the substrate concentration, Lu et al. (2013) found that it can improve the yield and concentration of ethanol without affecting the conversion of cellulose to ethanol.

##### CBP process

As mentioned above, generally, the conversion of lignocellulosic biomass to ethanol involves three main steps, pretreatment, enzymatic hydrolysis, and ethanol fermentation. The integration of these steps is very important for simplifying the process and reducing the production cost of cellulosic ethanol. CBP is a less energy-intensive and potentially low-cost process to produce cellulosic ethanol and other important industrial products (Agbor et al. 2014; Han et al. 2018). CBP has the potential to combine and simplify multiple processing steps, including cellulase production, enzymatic hydrolysis and bioconversion, into a one-step operation, that is, a single microorganism can fulfill hydrolytic enzymes production, polysaccharides hydrolysis, and ethanol fermentation. Compared with SHF and SSF, the CBP process can be conducted in a single vessel at a single optimized temperature, simplifying the pilot test and process development. In addition, in the CBP process, less pretreatment of lignocellulosic biomass is required, all of these keep the production cost of cellulosic bioethanol at a very low level. However, the process also results in significant by-product formation and low tolerance to ethanol. At present, there is no natural microorganism that can produce cellulosic ethanol through this CBP strategy. It is necessary to develop suitable engineered strains through artificial modification. There are two directions for the modification of CBP engineered strains: (1) engineering cellulase producers to make them have the ability to produce ethanol; (2) engineering ethanol producers to make them produce cellulase (Liu et al. 2019). Although the CBP is suitable for producing high-value products and low-cost fuels, the engineered microorganisms used in CBP are still immature compared with traditional processes, limiting its industrial application.

## Other value-added products

In addition to ethanol production, corn fiber can also be used to produce a variety of high value-added products through different processing technologies, the products include xylitol, corn fiber gum (CFG), corn fiber oil, dietary fiber (DF), etc.

### Dietary fiber

Dietary fiber is usually defined as a macromolecule present in the diet that is resistant to digestion by human endogenous enzymes. It is mainly composed of plant cell wall residues, such as cellulose, hemicelluloses, pectin polysaccharides, and lignin (Oomah et al. 2011). According to the solubility in water, dietary fiber is divided into two categories, namely, soluble dietary fiber and insoluble dietary fiber. Dietary fiber plays an important role in improving health through disease prevention and control, for example, improving intestinal function, lowering cholesterol, and increasing microbial biomass. Due to these health benefits, dietary fiber is a major part of the market for functional foods. Dietary fiber mainly exists in beans, grains, vegetables, etc., and corn fiber with high fiber content is one of the important sources of dietary fiber in grains. Wang et al. (2013a, b) used microwave-assisted extraction to produce dietary fiber from corn fiber, by optimizing extraction process conditions using response surface methodology analysis, including the optimal particle size of corn fiber (mesh size 40), the ratio of liquid to solid (25 mL/g), ultrasonic power (180 W) and ultrasonic time (80 min), the maximum yield of dietary fiber reached 86.84%.

### Corn fiber gum (CFG)

CFG is a soluble and highly branched arabinoxylan with low viscosity, high molar mass, and good emulsifying performance. It could be obtained by extracting corn fiber with alkaline hydrogen peroxide and ethanol precipitation (Yadav et al. 2007). Since hydrophobic proteins and lipids are combined with the carbohydrate moiety in CFG, CFG shows the potential as an emulsifier for oil-in-water emulsion systems. This allows CFG to be used as a substitute for gum Arabic in adhesives, thickeners, stabilizers, and emulsifiers. Yadav et al. (2010) covalently combined CFG with lactoglobulin and whey protein isolates to form a complex, compared to CFG or protein alone, the emulsification stability of the complex was significantly improved. In addition, due to the nutritional phenolic compounds in CFG chains, CFG also exhibits unique biological activities, such as antioxidant activity, fermentability, and immunomodulatory properties. Singkhornart et al. (2013) studied the effect of the combination of extrusion and alkali pretreatment on the extraction of CFG and found that extrusion conditions (moisture content and screw speed) affected the yield of CFG. The association between extrusion and sodium hydroxide pretreatment improved the fractionation of the corn fiber hemicelluloses polymer and increased the CFG yield by 12%. With the expansion of the market base, the new uses of CFG can be extended to snacks, baked goods, beverages, specialty foods, edible coatings, supplements, and other fields.

### Corn fiber oil

Corn fiber oil contains phytosterols that lower serum LDL-cholesterol levels. There are three main phytosterols in corn fiber oil: ferulate phytosterol esters, free phytosterols, and fatty acyl phytosterol esters. Because of the low oil content in corn fiber, only 1.5–3% of the weight, increasing the concentrations of oil and phytosterols before extraction can significantly reduce the extraction cost. Singh et al. (2003) investigated the effects of different treatments on removing non-lipid components (polysaccharides) from corn fibers. The results showed that the oil concentration in the residual solids increased from 1.4 to 12.2%, and the total phytosterols level increased from 176.9 to 1433.1 mg/g after treatment with sulfuric acid at 121 °C compared with untreated corn fiber. In addition, it was also shown that the oil and phytosterols in the residue remained intact. At present, the extraction of corn fiber oil is usually combined with the production of corn fiber ethanol, and corn fiber oil is extracted from saccharified corn fiber residue to maximize the use of resources and increase revenue.

### Xylitol

Xylitol is an expensive natural sweetener, and its sweetness is equivalent to sucrose but does not cause tooth decay and is safe for diabetic patients (Rose et al. 2010). Xylitol is mostly produced by hydrotreating xylose, and corn fiber with high xylan content is a promising raw material. Xylitol is also produced by the biosynthesis method using some microorganisms. Although many microorganisms can produce xylitol from xylose in corn fiber hydrolysate, glucose and inhibitors in the hydrolysate may inhibit the use of xylose by yeast and the growth of yeast. To reduce the inhibition of glucose, Leathers and Dien (2000) produced xylitol from sugar mixtures through a two-stage fermentation process. After the glucose was consumed, yeast cells were taken from the mixed sugar culture and replaced with cells grown in the xylose culture alone, and ultimately about 27% of the xylose in corn fiber hydrolysate was converted to xylitol. Rao et al. (2006) studied the production of xylitol from corn fiber hydrolysates by *Candida tropicalis*. However, due to inhibitors in the hydrolysate, the xylitol yield was low (0.43 g/g xylose). To reduce the influence of inhibitors in hydrolysate on yeast and improve the production of xylitol, the yeast cells were first grown on the corn fiber hydrolysate supplemented with certain nutrients. After growing to a certain stage, the cells were recovered by centrifugation and resuspended in the same fresh hydrolysate, and the whole adaptation was continued for 25 cycles. After adaptation, xylitol yield reached 0.58 g/g xylose with corn fiber hydrolysate.

After producing products, such as ethanol, xylitol, corn fiber gum, corn fiber oil, and dietary fiber, corn fiber can also be used to produce other products through biorefining. For example, the production of corn fiber gum by alkali treatment can simultaneously release the ferulic acid in the corn fiber into the solution, and it could be obtained by separating the corn fiber gum through ethanol precipitation (Saulnier et al. 1995). In addition, Singh et al. (2003) found that by hydrolyzing and removing some non-oil components (such as starch, cellulose, and hemicellulose), the content of oil and phytosterol in corn fiber can be effectively increased. This can be combined with ethanol production, where enzymes are used to convert carbohydrates into monosaccharides, and the remainder is used to produce corn fiber oil. At the same time, in the current ethanol production, glucose and xylose are mainly used, and l-arabinose can be separated from the fermentation broth, which has a good health care function.

## Perspectives

As the advantages of corn fiber as raw material, such as high reserves and low costs of transportation and collection, it will be of great benefit to the production of cellulosic ethanol if the corn fiber can be economically converted into ethanol, in particular, if it is combined with the existing ethanol production process with corn as a feedstock. (Bothast and Schlicher 2005; Nair et al. 2017). To realize the combination of the two processes, (Li et al. 2019) developed an in-situ acid pretreatment method in the distillation process and integrated it into the dry grinding process to recycle the pretreated stillage to the liquefication stage, which can effectively improve the yield of ethanol. In addition, by developing cellulases with tolerances of high sugar concentration and high-temperature, and appropriately cooling the starch liquefaction process, corn fiber and corn starch can be hydrolyzed and saccharified simultaneously. Another method that combining corn ethanol production process is that, in the grind stage, corn fiber can be separated by a wet milling process for separate enzymatic hydrolysis, and then the enzymatic hydrolysis solution can be added to the mash for simultaneous fermentation.

At present, the production of ethanol using corn fiber has been applied in many corn ethanol plants in the United States, and the ethanol yield has generally increased by more than 5%. Unlike other lignocellulosic materials, corn fiber is rich in hemicelluloses with a complex structure, accounting for about 40% of corn fiber. How to break the hemicelluloses barrier is very important for high-efficiency hydrolyzing corn fiber into fermentable sugars using enzyme. On the other hand, the enzyme cost for corn fiber ethanol production is still high, and the complex structure of hemicelluloses makes the degradation of corn fiber requires a very complex enzyme system. However, the commercial enzymes on the market are not ideal for the degradation of corn fiber, and it is necessary to continue developing targeted enzyme preparations. The complex sugars compositions in the enzymatic hydrolysates of corn fiber require the strains capable of fermenting hexose and pentose simultaneously. There are still many works to be done in the field on the existing basis. For example, (1) optimizing suitable pretreatment process and process parameters to reduce inhibitors and devices cost; (2) developing an enzyme preparation that could hydrolyze corn fiber more efficiently according to the chemical components and structural characteristics of corn fiber to reduce enzyme dosage in enzymatic hydrolysis; (3) high-efficiency production of enzyme by selecting appropriate enzyme-producing strains and strain modification, and optimizing fermentation process for producing low-cost enzyme; (4) modifying the fermentation strain by various biotechnologies including metabolic engineering, protein engineering and genome technology to simultaneously convert hexoses and pentoses in hydrolysates efficiently and to enhance the tolerance of strain to different toxic substances; (5) embedding corn fiber ethanol production into conventional corn ethanol process to reduce investment and production costs; (6) combining the production of value-added products with the production of corn fiber ethanol to improve economic efficiency.

## Data Availability

Not applicable.

## Abbreviations

DDGS: Distillers dried grains with solubles
GAX: Glucuronoarabinoxlyan
HMF: Hydroxymethylfurfural
PS: Particle size
AFEX: Ammonia fiber explosion
LHW: Liquid hot water
SHF: Separate hydrolysis and fermentation
SSF: Simultaneous saccharification and fermentation
S-SSF: Semi-simultaneous saccharification and fermentation
CBP: Consolidated bioprocessing
LPMOs: Lytic polysaccharide monooxygenases
GFOR: Glucose–fructose oxidoreductase
CFG: Corn fiber gum
DF: Dietary fiber

## References

[CR1] Agbor V, Carere C, Cicek N, Sparling R, Levin D, Waldron K (2014). Biomass pretreatment for consolidated bioprocessing (CBP). Advances in biorefineries: biomass waste supply chain exploit.

[CR2] Allen SG, Schulman D, Lichwa J, Antal MJ, Laser M, Lynd LR (2001). A comparison between hot liquid water and steam fractionation of corn fiber. Ind Eng Chem Res.

[CR3] Alvira P, Tomás-Pejó E, Ballesteros M, Negro MJ (2010). Pretreatment technologies for an efficient bioethanol production process based on enzymatic hydrolysis: a review. Bioresour Technol.

[CR4] Bajpai P, Bajpai P (2016). Structure of lignocellulosic biomass. Pretreatment of lignocellulosic biomass for biofuel production.

[CR5] Balat M, Balat H, Öz C (2008). Progress in bioethanol processing. Prog Energy Combust Sci.

[CR6] Behera S, Arora R, Nandhagopal N, Kumar S (2014). Importance of chemical pretreatment for bioconversion of lignocellulosic biomass. Renew Sust Energ Rev.

[CR7] Beri D, York WS, Lynd LR, Peña MJ, Herring CD (2020). Development of a thermophilic coculture for corn fiber conversion to ethanol. Nat Commun.

[CR8] Bertrand E, Pradel M, Dussap CG, Soccol CR, Brar SK, Faulds C, Ramos LP (2016). Economic and environmental aspects of biofuels. Green fuels technology.

[CR9] Bhutto AW, Qureshi K, Harijan K, Abro R, Abbas T, Bazmi AA, Karim S, Yu G (2017). Insight into progress in pre-treatment of lignocellulosic biomass. Energy.

[CR10] Bischof RH, Ramoni J, Seiboth B (2016). Cellulases and beyond: the first 70 years of the enzyme producer *Trichoderma reesei*. Microb Cell Factories.

[CR11] Bothast RJ, Schlicher MA (2005). Biotechnological processes for conversion of corn into ethanol. Appl Microbiol Biotechnol.

[CR12] Bura R, Mansfield SD, Saddler JN, Bothast RJ (2002). SO_2_-catalyzed steam explosion of corn fiber for ethanol production. Appl Biochem Biotechnol.

[CR13] Bura R, Bothast RJ, Mansfield SD, Saddler JN (2003). Optimization of SO_2_-catalyzed steam pretreatment of corn fiber for ethanol production. Appl Biochem Biotechnol.

[CR14] Byadgi SA, Kalburgi PB (2016). Production of bioethanol from waste newspaper. Proc Env Sci.

[CR15] Cai Z, Zhang B, Li Y (2012). Engineering *Saccharomyces cerevisiae* for efficient anaerobic xylose fermentation: reflections and perspectives. Biotechnol J.

[CR16] Cairns TC, Nai C, Meyer V (2018). How a fungus shapes biotechnology: 100 years of *Aspergillus niger* research. Fungal Biol Biotechnol.

[CR17] Chatterjee S, Sharma S, Prasad RK, Datta S, Dubey D, Meghvansi MK, Vairale MG, Veer V (2015). Cellulase enzyme based biodegradation of cellulosic materials: an overview. S A J Exp Biol.

[CR18] Chen H (2014). Chemical composition and structure of natural lignocellulose. Biotechnol Lignocellul.

[CR19] Chong H, Huang L, Yeow J, Wang I, Zhang H, Song H, Jiang R (2013). Improving ethanol tolerance of *Escherichia coli* by rewiring its global regulator cAMP receptor protein (CRP). PLoS ONE.

[CR20] Conde-Mejía C, Jiménez-Gutiérrez A, El-Halwagi M (2012). A comparison of pretreatment methods for bioethanol production from lignocellulosic materials. Process Saf Environ Prot.

[CR21] Coutinho PM, Andersen MR, Kolenova K, vanKuyk PA, Benoit I, Gruben BS, Trejo-Aguilar B, Visser H, van Solingen P, Pakula T, Seiboth B, Battaglia E, Aguilar-Osorio G, de Jong JF, Ohm RA, Aguilar M, Henrissat B, Nielsen J, Stålbrand H, de Vries RP (2009). Post-genomic insights into the plant polysaccharide degradation potential of *Aspergillus nidulans* and comparison to *Aspergillus niger* and *Aspergillus oryzae*. Fungal Genet Biol.

[CR22] de Gonzalo G, Colpa DI, Habib MHM, Fraaje MW (2016). Bacterial enzymes involved in lignin degradation. I Biotechnol.

[CR23] Deanda K, Zhang M, Eddy C, Picataggio S (1996). Development of an arabinose-fermenting *Zymomonas mobilis* strain by metabolic pathway engineering. Appl Environ Microbiol.

[CR24] Dien BS, Cotta MA, Jeffries TW (2003). Bacteria engineered for fuel ethanol production: current status. Appl Microbiol Biotechnol.

[CR25] Druzhinina IS, Kubicek CP (2016). Familiar Stranger: Ecological genomics of the model saprotroph and industrial enzyme producer *Trichoderma reesei* breaks the stereotypes. Adv Appl Microbiol.

[CR26] Druzhinina IS, Kubicek CP (2017). Genetic engineering of *Trichoderma reesei* cellulases and their production. Microb Biotechnol.

[CR27] Druzhinina IS, Chenthamara K, Zhang J, Atanasova L, Yang D, Miao Y, Rahimi MJ, Grujic M, Cai F, Pourmehdi S, Salim KA, Pretzer C, Kopchinskiy AG, Henrissat B, Kuo A, Hundley H, Wang M, Aerts A, Salamov A, Lipzen A, LaButti K, Barry K, Grigoriev IV, Shen Q, Kubicek CP (2018). Massive lateral transfer of genes encoding plant cell wall-degrading enzymes to the mycoparasitic fungus *Trichoderma* from its plant-associated hosts. PLoS Genet.

[CR28] Du J, Song W, Zhang X, Zhao J, Liu G, Qu Y (2018). Differential reinforcement of enzymatic hydrolysis by adding chemicals and accessory proteins to high solid loading substrates with different pretreatments. Bioprocess Biosyst Eng.

[CR29] Dunn KL, Rao CV (2014). Expression of a xylose-specific transporter improves ethanol production by metabolically engineered *Zymomonas mobilis*. Appl Microbiol Biotechnol.

[CR30] Eylen DV, Dongen F, Kabel M, Bont J (2011). Corn fiber, cobs and stover: enzyme-aided saccharification and co-fermentation after dilute acid pretreatment. Bioresour Technol.

[CR31] Feng X, Yao Y, Xu N, Jia H, Li X, Zhao J, Chen C, Qu Y (2021). Pretreatment affects profits from xylanase during enzymatic saccharification of corn stover through changing the interaction between lignin and xylanase protein. Front Microbiol.

[CR32] Galbe M, Wallberg O, Zacchi G, Young M (2011). Techno-economic aspects of ethanol production from lignocellulosic agricultural crops and residues. Comprehensive biotechnology.

[CR33] Gao J, Qian Y, Wang Y, Qu Y, Zhong Y (2017). Production of the versatile cellulase for cellulose bioconversion and cellulase inducer synthesis by genetic improvement of *Trichoderma reesei*. Biotechnol Biofuels.

[CR34] Gao M, Ploessl D, Shao Z (2019). Enhancing the co-utilization of biomass-derived mixed sugars by yeasts. Front Microbiol.

[CR35] Gao L, He X, Guo Y, Wu Z, Zhao J, Liu G, Qu Y (2021). Combinatorial engineering of transcriptional activators in *Penicillium oxalicum* for improved production of corn-fiber-degrading enzymes. J Agric Food Chem.

[CR36] Gong W, Zhang H, Liu S, Zhang L, Gao P, Chen G, Wang L (2015). Comparative secretome analysis of *Aspergillus niger*, *Trichoderma reesei*, and *Penicillium oxalicum* during solid-state fermentation. Appl Biochem Biotechnol.

[CR37] Granados-Arvizu JA, Amaro-Reyes A, García-Almendárez BE, Gracida-Rodríguez JN, Regalado C (2017). Optimization of dilute acid pretreatment of corn pericarp by response surface methodology. BioResources.

[CR38] Guo Y, Huang J, Xuo N, Jia H, Li X, Zhao J, Qu Y (2022). A detoxification-free process for enhanced ethanol production from corn fiber under semi-simultaneous saccharification and fermentation. Front Microbiol.

[CR39] Han X, Liu G, Pan Y, Song W, Qu Y (2018). Consolidated bioprocessing for sodium gluconate production from cellulose using *Penicillium oxalicum*. Bioresour Technol.

[CR40] Hanchar RJ, Teymouri F, Nielson CD, McCalla D, Stowers MD (2007). Separation of glucose and pentose sugars by selective enzyme hydrolysis of AFEX-treated corn fiber. Appl Biochem Biotechnol.

[CR41] Harris PV, Xu F, Kreel NE, Kang C, Fukuyama S (2014). New enzyme insights drive advances in commercial ethanol production. Curr Opin Chem Biol.

[CR42] Harun MY, Radiah ABD, Abidin ZZ, Yunus R (2011). Effect of physical pretreatment on dilute acid hydrolysis of water hyacinth (*Eichhornia*
*crassipes*). Bioresour Technol.

[CR43] Hendriks ATWM, Zeeman G (2009). Pretreatments to enhance the digestibility of lignocellulosic biomass. Bioresour Technol.

[CR44] Horn SJ, Vaaje-Kolstad G, Westereng B, Eijsink VGH (2012). Novel enzymes for the degradation of cellulose. Biotechnol Biofuels.

[CR45] Ibraheem O, Ndimba BK (2013). Molecular adaptation mechanisms employed by ethanologenic bacteria in response to lignocellulose-derived inhibitory compounds. Int J Biol Sci.

[CR46] Jeong D, Oh EJ, Ko JK, Nam JO, Park HS, Jin YS, Lee EJ (2020). Metabolic engineering considerations for the heterologous expression of xylose-catabolic pathways in *Saccharomyces cerevisiae*. PLoS ONE.

[CR47] Jönsson LJ, Martín C (2016). Pretreatment of lignocellulose: formation of inhibitory by-products and strategies for minimizing their effects. Bioresour Technol.

[CR48] Juneja A, Noordam B, Pel H, Basu R, Appeldoorn M, Singh V (2021). Optimization of two-stage pretreatment for maximizing ethanol production in 1.5G technology. Bioresour Technol.

[CR49] Juturu V, Wu JC (2014). Microbial exo-xylanases: a mini review. Appl Biochem Biotechnol.

[CR50] Kameshwar AKS, Qin W (2018). Structural and functional properties of pectin and lignin–carbohydrate complexes de-esterases: a review. Bioresour Bioprocess.

[CR51] Kang J, Guo Q, Shi Y (2019). NMR and methylation analysis of hemicellulose purified from corn bran. Food Hydrocoll.

[CR52] Kim D, Orrego D, Ximenes EA, Ladisch MR (2017). Cellulose conversion of corn pericarp without pretreatment. Bioresour Technol.

[CR53] Kuhad RC, Gupta R, Singh A (2011). Microbial cellulases and their industrial applications. Enzyme Res.

[CR54] Kurambhatti CV, Kumar D, Rausch KD, Tumbleson ME, Singh V (2018). Increasing ethanol yield through fiber conversion in corn dry grind process. Bioresour Technol.

[CR55] Kwak S, Jin Y (2017). Production of fuels and chemicals from xylose by engineered *Saccharomyces*
*cerevisiae*: a review and perspective. Microb Cell Fact.

[CR56] Leathers TD, Dien BS (2000). Xylitol production from corn fibre hydrolysates by a two-stage fermentation process. Process Biochem.

[CR57] Lennartsson PR, Erlandsson P, Taherzadeh MJ (2014). Integration of the first and second generation bioethanol processes and the importance of by-products. Bioresour Technol.

[CR58] Li X, Lu J, Zhao J, Qu Y (2014). Characteristics of corn stover pretreated with liquid hot water and fed-batch semi-simultaneous saccharification and fermentation for bioethanol production. PLoS ONE.

[CR59] Li H, Shen Y, Wu M, Hou J, Jiao C, Li Z, Liu X, Bao X (2016). Engineering a wild-type diploid *Saccharomyces cerevisiae* strain for second-generation bioethanol production. Bioresour Bioprocess.

[CR60] Li X, Xu Z, Yu J, Huang H, Jin M (2019) In situ pretreatment during distillation improves corn fiber conversion and ethanol yield in the dry mill process. Green Chem 21(5), 1080-1090. 10.1039/C8GC03447H

[CR61] Li Z, Liu G, Qu Y (2017). Improvement of cellulolytic enzyme production and performance by rational designing expression regulatory network and enzyme system composition. Bioresour Technol.

[CR62] Lin M, Ryu GH (2014). Effects of thermomechanical extrusion and particle size reduction on bioconversion rate of corn fiber for ethanol production. Cereal Chem.

[CR63] Liu G, Qu Y (2019). Engineering of filamentous fungi for efficient conversion of lignocellulose: tools, recent advances and prospects. Biotechnol Adv.

[CR64] Liu G, Qin Y, Li Z, Qu Y (2013). Improving lignocellulolytic enzyme production with *Penicillium*: from strain screening to systems biology. Biofuels.

[CR65] Liu G, Zhang L, Wei X, Zou G, Qin Y, Ma L, Li J, Zheng H, Wang S, Wang C, Xun L, Zhao GP, Zhou Z, Qu Y (2013). Genomic and secretomic analyses reveal unique features of the lignocellulolytic enzyme system of *Penicillium decumbens*. PLoS ONE.

[CR66] Liu ZH, Qin L, Zhu JQ, Li BZ, Yuan YJ (2014). Simultaneous saccharification and fermentation of steam-exploded corn stover at high glucan loading and high temperature. Biotechnol Biofuels.

[CR67] Liu G, Zhang J, Bao J (2016). Cost evaluation of cellulase enzyme for industrial-scale cellulosic ethanol production based on rigorous Aspen Plus modeling. Bioprocess Biosyst Eng.

[CR68] Liu CG, Xiao Y, Xia XX, Zhao XQ, Peng L, Srinophakun P, Bai FW (2019). Cellulosic ethanol production: progress, challenges and strategies for solutions. Biotechnol Adv.

[CR69] Lovett JC, Hards S, Clancya J, Snell C (2011). Multiple objectives in biofuels sustainability policy. Energy Environ Sci.

[CR70] Lu J, Li X, Yang R, Yang L, Zhao J, Liu Y, Qu Y (2013). Fed-batch semi-simultaneous saccharification and fermentation of reed pretreated with liquid hot water for bio-ethanol production using *Saccharomyces cerevisiae*. Bioresour Technol.

[CR71] Lu X, Feng X, Li X, Zhao J (2018). The adsorption properties of endoglucanase to lignin and their impact on hydrolysis. Bioresour Technol.

[CR72] Mankar AR, Pandey A, Modak A, Pant KK (2021). Pretreatment of lignocellulosic biomass: a review on recent advances. Bioresour Technol.

[CR73] Marulanda VA, Gutierrez CDB, Alzate CAC (2019). Thermochemical, biological, biochemical, and hybrid conversion methods of bio-derived molecules into renewable fuels. Advanced bioprocessing for alternative fuels, biobased chemicals and bioproducts: technologies and approaches for scale-up and commercialization.

[CR74] Matsuzawa T, Kameyama A, Yaoi K (2020). Identification and characterization of α-xylosidase involved in xyloglucan degradation in *Aspergillus*
*oryzae*. Appl Microbiol Biotechnol.

[CR75] Méndez-Líter J, de Eugenio L, Domínguez M, Prieto A, Martinez M (2021). Hemicellulases from *Penicillium* and *Talaromyces* for lignocellulosic biomass valorization: a review. Bioresour Technol.

[CR76] Meng X, Ma L, Li T, Zhu H, Guo K, Liu D, Ran W, Shen Q (2020). The functioning of a novel protein, Swollenin, in promoting the lignocellulose degradation capacity of *Trichoderma guizhouense* NJAU4742 from a proteomic perspective. Bioresour Technol.

[CR77] Menon R, Gonzalez T, Ferruzzi M, Jackson E, Winderl D, Watson J (2016). Oats—from farm to fork. Advances in food and nutrition research.

[CR78] Mezzina MP, Álvarez DS, Egoburo DE, Díaz Peña R, Nikel PI, Pettinari MJ (2017). A new player in the biorefineries field: Phasin PhaP enhances tolerance to solvents and boosts ethanol and 1,3-propanediol synthesis in *Escherichia coli*. Appl Environ Microbiol.

[CR79] Moore R, Thornhill K, Weinzierl B, Sauer D, D’Ascoli E, Kim J, Lichtenstern M, Scheibe M, Beaton B, Beyersdorf AJ, Barrick J, Bulzan D, Corr CA, Crosbie E, Jurkat T, Martin R, Riddick D, Shook M, Slover G, Voigt C, White R, Winstead E, Yasky R, Ziemba LD, Brown A, Schlager H, Anderson BE (2017). Show fewer authors biofuel blending reduces particle emissions from aircraft engines at cruise conditions. Nature.

[CR80] Moysés DN, Reis VC, de Almeida JR, de Moraes LM, Torres FA (2016). Xylose fermentation by *Saccharomyces cerevisiae*: challenges and prospects. Int J Mol Sci.

[CR81] Mussatto SI, Dragone G, Guimaraes PMR, Paulo J, Silva A, Carneiro LM, Roberto IC, Vicente A, Domingues L, Teixeira JA (2010). Technological trends, global market, and challenges of bio-ethanol production. Biotechnol Adv.

[CR82] Myat L, Ryu GH (2014). Thermo-mechanical extrusion and sodium hydroxide pretreatments for ethanol production from destarched corn fiber. Environ Prog Sustain Energy.

[CR83] Nair RB, Kalif M, Ferreira JA, Taherzadeh MJ, Lennartsson PR (2017). Mild-temperature dilute acid pretreatment for integration of first and second generation ethanol processes. Bioresour Technol.

[CR84] Nigam PS, Singh A (2011). Production of liquid biofuels from renewable resources. Prog Energy Combust Sci.

[CR85] Noureddini H, Byun J (2010). Dilute-acid pretreatment of distillers’ grains and corn fiber. Bioresour Technol.

[CR86] Ofosu-Appiah C, Zakpaa HD, Mak-Mensah E, Bentil JA (2016). Evaluation of ethanol production from pito mash using *Zymomonas*
*mobilis* and *Saccharomyces*
*cerevisiae*. Afr J Biotechnol.

[CR87] Ohta K, Beall DS, Mejia JP, Shanmugam KT, Ingram LO (1991). Genetic improvement of *Escherichia coli* for ethanol production: chromosomal integration of *Zymomonas mobilis* genes encoding pyruvate decarboxylase and alcohol dehydrogenase II. Appl Environ Microbiol.

[CR88] Oliveira DM, Mota TR, Ooliva B, Segato F, Marchiosi R, Ferrarese-Filho O, Faulds CB, dos Santos WD (2019). Feruloyl esterases: biocatalysts to overcome biomass recalcitrance and for the production of bioactive compounds. Bioresour Technol.

[CR89] Oomah B, Patras A, Rawson A, Singh N, Compos-Vega R, Tiwari BK, Gowen A, McKenna B (2011). Chemistry of pulses. Pulse foods: processing, quality and nutraceutical applications.

[CR90] Poria V, Saini JK, Singh S, Nain L, Kuhad RC (2020). Arabinofuranosidases: characteristics, microbial production, and potential in waste valorization and industrial applications. Bioresour Technol.

[CR91] Pragya N, Pandey KK, Sahoo PK (2013). A review on harvesting, oil extraction and biofuels production technologies from microalgae. Renew Sust Energ Rev.

[CR92] Puri M, Abraham RE, Barrow CJ (2012). Biofuel production: prospects, challenges and feedstock in Australia. Renew Sust Energ Rev.

[CR93] Qian Y, Zhong L, Gao J, Sun N, Wang Y, Sun G, Qu Y, Zhong Y (2017). Production of highly efficient cellulase mixtures by genetically exploiting the potentials of *Trichoderma*
*reesei* endogenous cellulases for hydrolysis of corncob residues. Microb Cell Factories.

[CR94] Qu J, Zhu J, Liu G, Qu Y, Lübeck M (2018). Identification of key components for the optimization of cellulase mixtures using a proteomic strategy. Cellulases methods in molecular biology.

[CR95] Qureshi N, Ezeji TC, Ebener J, Dien BS, Cotta MA, Blaschek HP (2008). Butanol production by *Clostridium*
*beijerinckii*. Part I: use of acid and enzyme hydrolyzed corn fiber. Bioresour Technol.

[CR96] Rao RS, Jyothi CP, Prakasham RS, Sarma PN, Rao LV (2006). Xylitol production from corn fiber and sugarcane bagasse hydrolysates by *Candida tropicalis*. Bioresour Technol.

[CR97] Robak K, Balcerek M (2020). Current state-of-the-art in ethanol production from lignocellulosic feedstocks. Microbiol Res.

[CR98] Rocha MH, Capaz RS, Lora EES, Nogueira LAH, Leme MMV, Renó MLG, Olmo OA (2014). Life cycle assessment (LCA) for biofuels in Brazilian conditions: a meta-analysis. Renew Sust Energ Rev.

[CR99] Roche CM, Loros JJ, McCluskey K, Glass NL (2014). *Neurospora crassa*: looking back and looking forward at a model microbe. Am J Bot.

[CR100] Rose DJ, Inglett GE, Liu SX (2010). Utilisation of corn (*Zea mays*) bran and corn fiber in the production of food components. J Sci Food Agric.

[CR101] Saha BC (2003). Hemicellulose bioconversion. J Ind Microbiol Biotechnol.

[CR102] Saha BC, Cotta MA (2010). Comparison of pretreatment strategies for enzymatic saccharification and fermentation of barley straw to ethanol. New Biotechnol.

[CR103] Saini JK, Saini R, Tewari L (2015). Lignocellulosic agriculture wastes as biomass feedstocks for second-generation bioethanol production: concepts and recent developments. 3 Biotech.

[CR104] Sanny T, Arnaldos M, Kunkel SA, Pagilla KR, Stark BC (2010). Engineering of ethanolic *E. coli* with the *Vitreoscilla* hemoglobin gene enhances ethanol production from both glucose and xylose. Appl Microbiol Biotechnol.

[CR105] Saulnier L, Marot C, Chanliaud E, Thibault J (1995). Cell wall polysaccharide interactions in maize bran. Carbohydr Polym.

[CR106] Scheller HV, Ulvskov P (2010). Hemicelluloses. Annu Rev Plant Biol.

[CR107] Searchinger TD, Wirsenius S, Beringer T, Dumas P (2018). Assessing the efficiency of changes in land use for mitigating climate change. Nature.

[CR108] Sharma P, Gujral HS, Singh B (2012). Antioxidant activity of barley as affected by extrusion cooking. Food Chem.

[CR109] Sharma B, Larroche C, Dussap CG (2020). Comprehensive assessment of 2G bioethanol production. Bioresour Technol.

[CR110] Shen J, Agblevor FA (2010). Modeling semi-simultaneous saccharification and fermentation of ethanol production from cellulose. Biomass Bioenergy.

[CR111] Shrestha P, Rasmussen M, Khanal SK, Pometto AL, van Leeuwen JH (2008). Solid-substrate fermentation of corn fiber by *Phanerochaete*
*chrysosporium* and subsequent fermentation of hydrolysate into ethanol. J Agric Food Chem.

[CR112] Shrestha P, Khanal SK, Pometto AL, van Leeuwen JH (2009). Enzyme production by wood-rot and soft-rot fungi cultivated on corn fiber followed by simultaneous saccharification and fermentation. J Agric Food Chem.

[CR113] Shrestha P, Khanal SK, Pometto AL, Hans van Leeuwen J (2010). Ethanol production via in situ fungal saccharification and fermentation of mild alkali and steam pretreated corn fiber. Bioresour Technol.

[CR114] Sims REH, Mabee W, Saddler JN, Taylor M (2010). An overview of second generation biofuel technologies. Bioresour Technol.

[CR115] Singh V, Johnston DB, Moreau RA, Hicks KB, Dien BS, Bothast RJ (2003). Pretreatment of wet-milled corn fiber to improve recovery of corn fiber oil and phytosterols. Cereal Chem.

[CR116] Singh P, Suman A, Tiwari P, Arya N, Gaur A, Shrivastava AK (2008). Biological pretreatment of sugarcane trash for its conversion to fermentable sugars. World J Microbiol Biotechnol.

[CR117] Singhania RR, Dixit P, Patel AK, Giri BS, Kuo CH, Chen CW, Dong CD (2021). Role and significance of lytic polysaccharide monooxygenases (LPMOs) in lignocellulose deconstruction. Bioresour Technol.

[CR118] Singkhornart S, Lee SG, Ryu GH (2013). Influence of twin-screw extrusion on soluble arabinoxylans and corn fiber gum from corn fiber. J Sci Food Agric.

[CR119] Sun J, Tian C, Diamond S, Glass NL (2012). Deciphering transcriptional regulatory mechanisms associated with hemicellulose degradation in *Neurospora*
*crassa*. Eukaryot Cell.

[CR120] Taherzadeh MJ, Karimi K (2008). Pretreatment of lignocellulosic wastes to improve ethanol and biogas production: a review. Int J Mol Sci.

[CR121] Vohra M, Manwar J, Manmode R, Padgilwar S, Patil S (2014). Bioethanol production: feedstock and current technologies. J Environ Chem Eng.

[CR122] Wang Z, Chen M, Xu Y, Li S, Lu W, Ping S, Zhang W, Lin M (2008). An ethanol-tolerant recombinant *Escherichia coli* expressing *Zymomonas mobilis pdc* and *adhB* genes for enhanced ethanol production from xylose. Biotechnol Lett.

[CR123] Wang A, Wu L, Li X (2013). Optimization of ultrasonic-assisted preparation of dietary fiber from corn pericarp using response surface methodology. J Sci Food Agric.

[CR124] Wang C, Liu C, Hong J, Zhang K, Ma Y, Zou S, Zhang M (2013). Unmarked insertional inactivation in the *gfo* gene improves growth and ethanol production by *Zymomonas mobilis* ZM4 in sucrose without formation of sorbitol as a by-product, but yields opposite effects in high glucose. Biochem Eng J.

[CR125] Wang X, Gao Q, Bao J (2017). Enhancement of furan aldehydes conversion in *Zymomonas*
*mobilis* by elevating dehydrogenase activity and cofactor regeneration. Biotechnol Biofuels.

[CR126] Wyman CE, Dale BE, Elander RT, Holtzapple M, Ladisch MR, Lee YY (2005). Coordinated development of leading biomass pretreatment technologies. Bioresour Technol.

[CR127] Xia J, Yang Y, Liu C, Yang S, Bai F (2019). Engineering *Zymomonas mobilis* for robust cellulosic ethanol production. Trends Biotechnol.

[CR128] Yadav MP, Johnston DB, Hotchkiss AT, Hicks KB (2007). Corn fiber gum: a potential gum Arabic replacer for beverage flavor emulsification. Food Hydrocoll.

[CR129] Yadav MP, Parris N, Johnston DB, Onwulata CI, Hicks KB (2010). Corn fiber gum and milk protein conjugates with improved emulsion stability. Carbohydr Polym.

[CR130] Yang Y, Yang J, Liu J, Wang R, Liu L, Wang F, Yuan H (2018). The composition of accessory enzymes of *Penicillium*
*chrysogenum* P33 revealed by secretome and synergistic effects with commercial cellulase on lignocellulose hydrolysis. Bioresour Technol.

[CR131] Yao G, Wu R, Kan Q, Gao L, Liu M, Yang P, Du J, Li Z, Qu Y (2016). Production of a high-efficiency cellulase complex via β-glucosidase engineering in *Penicillium oxalicum*. Biotechnol Biofuels.

[CR132] Zhang M, Eddy C, Deanda K, Finkelstein M, Picataggio S (1995). Metabolic engineering of a pentose metabolism pathway in ethanologenic *Zymomonas mobilis*. Science.

[CR133] Zhang YHP, Himmel ME, Mielenz JR (2006). Outlook for cellulase improvement: screening and selection strategies. Biotechnol Adv.

[CR134] Zhang X, Shen Y, Shi W, Bao X (2010). Ethanolic cofermentation with glucose and xylose by the recombinant industrial strain *Saccharomyces cerevisiae* NAN-127 and the effect of furfural on xylitol production. Bioresour Technol.

[CR135] Zhang B, Zhan B, Bao J (2021). Reframing biorefinery processing chain of corn fiber for cellulosic ethanol production. Ind Crops Prod.

[CR136] Zhang W, Guo J, Wu X, Ren Y, Li C, Meng X, Liu W (2022). Reformulating the hydrolytic enzyme cocktail of *Trichoderma*
*reesei* by combining XYR1 overexpression and elimination of four major cellulases to improve saccharification of corn fiber. J Agric Food Chem.

[CR137] Zingaro KA, Papoutsakis ET (2013). GroESL overexpression imparts *Escherichia coli* tolerance to i-, n-, and 2-butanol, 1,2,4-butanetriol and ethanol with complex and unpredictable patterns. Metab Eng.

